# A portable system for processing donated whole blood into high quality components without centrifugation

**DOI:** 10.1371/journal.pone.0190827

**Published:** 2018-01-18

**Authors:** Sean C. Gifford, Briony C. Strachan, Hui Xia, Eszter Vörös, Kian Torabian, Taylor A. Tomasino, Gary D. Griffin, Benjamin Lichtiger, Fleur M. Aung, Sergey S. Shevkoplyas

**Affiliations:** 1 Halcyon Biomedical Incorporated, Friendswood, Texas, United States of America; 2 Department of Biomedical Engineering, University of Houston, Houston, Texas, United States of America; 3 Department of Laboratory Medicine, Section of Transfusion Medicine / Blood Donor Center, MD Anderson Cancer Center, Houston, Texas, United States of America; Institute for Transfusion Medicine and Immunohaematology Frankfurt, GERMANY

## Abstract

**Background:**

The use of centrifugation-based approaches for processing donated blood into components is routine in the industrialized world, as disparate storage conditions require the rapid separation of ‘whole blood’ into distinct red blood cell (RBC), platelet, and plasma products. However, the logistical complications and potential cellular damage associated with centrifugation/apheresis manufacturing of blood products are well documented. The objective of this study was to evaluate a proof-of-concept system for whole blood processing, which does not employ electromechanical parts, is easily portable, and can be operated immediately after donation with minimal human labor.

**Methods and findings:**

In a split-unit study (n = 6), full (~500mL) units of freshly-donated whole blood were divided, with one half processed by conventional centrifugation techniques and the other with the new blood separation system. Each of these processes took 2–3 hours to complete and were performed in parallel. Blood products generated by the two approaches were compared using an extensive panel of cellular and plasma quality metrics. Comparison of nearly all RBC parameters showed no significant differences between the two approaches, although the portable system generated RBC units with a slight but statistically significant improvement in 2,3-diphosphoglyceric acid concentration (p < 0.05). More notably, several markers of platelet damage were significantly and meaningfully higher in products generated with conventional centrifugation: the increase in platelet activation (assessed via P-selectin expression in platelets before and after blood processing) was nearly 4-fold higher for platelet units produced via centrifugation, and the release of pro-inflammatory mediators (soluble CD40-ligand, thromboxane B2) was significantly higher for centrifuged platelets as well (p < 0.01).

**Conclusion:**

This study demonstrated that a simple, passive system for separating donated blood into components may be a viable alternative to centrifugation—particularly for applications in remote or resource-limited settings, or for patients requiring highly functional platelet product.

## Introduction

The separation of a unit of whole blood (WB) into its constitutive components—red blood cells (RBCs), platelets, and plasma—soon after donation is required in order to maximize its clinical value to future transfusion recipients. Notwithstanding minor differences from region-to-region: processed units of packed RBCs (pRBCs) can be kept at 4–6°C for up to 6 weeks (given proper storage media), platelet concentrate (PC) is gently agitated at 20–22°C for up to 5–7 days, and plasma can be stored frozen (-18°C) for 12 months.[1, 2] The wide variance in optimal storage conditions between these three main blood components is the impetus for the complex logistics and labor-intensive workflow which currently accompany modern blood banking.

Nearly all of the >100 million individual blood components transfused each year worldwide [3] (>30 million in the U.S.[4]) are first produced by multi-stage centrifugation of freshly-donated WB units, or through (also centrifugation-based) apheresis collection of a larger amount of a specific component from a willing donor.[1] While centrifugation of blood allows for dense RBCs (~1.100 g/mL) to be reliably separated from platelets (~1.058 g/mL) and plasma (~1.026 g/mL),[1, 5] the approach nevertheless has well-documented disadvantages. Blood banking by this method is laborious, and high-capacity centrifuges represent major monetary commitments—both in capital expenditure and in operation/maintenance. WB units collected at mobile blood drives (which represent approximately two-thirds of all blood donations)[6] must be continually shuttled to a central facility for separation within the time frame set by FDA/AABB [7] to preserve the donor’s platelets (which are otherwise lost if WB is placed on ice) as well as sufficient plasma factor activity.[1] The complicated logistics of this process is a significant contributor to the operating costs of community and regional blood centers.[8]

Both the expense and complexity of centrifugation-based blood banking are typically prohibitive for low-income developing countries, where life-saving transfusions remain largely unavailable outside of select private hospitals and/or in cases where (often untested) WB is transfused from a suitable relative or paid donor.[3] Centrifugation-based WB processing is also not easily scalable, making it difficult to accommodate the influx of WB donations after mass casualty events.[9] Further, operation of high-capacity centrifuges requires a stable high-wattage power supply, rendering WB processing during natural disasters and blackouts unreliable and the availability of blood products unsustainable.[10]

In addition to concerns over their cost and availability, the quality of stored blood components is an issue increasingly at the forefront of transfusion medicine. A number of recent studies have highlighted the correlation between the length of time that units of RBCs or PC are stored following donation, and the subsequent clinical outcome of the patient receiving those products.[11–17] To what degree the reduction of component quality during storage can be attributed to cellular damage incurred by the high-speed (typically 3000–5000×*g*) centrifugation methods used to *initially process* units has not been well studied, as heretofore there has been no viable alternative methodology for comparison. However, these processes are more than adequate to trigger the natural, hemostatic response of platelets, as activation levels up to 50% are not uncommon.[18, 19]

To address the above limitations of conventional, centrifugation-based blood processing, several groups have explored alternative technologies, although each has reported notable drawbacks. Hollow fiber filtration is capable of extracting RBC product from WB, with minimal labor or equipment requirements, but requires relatively expensive membranes and results in diminished quality of plasma and a complete loss of the platelet population,[20–22] which is increasingly needed by trauma and oncology patients.[23] Single-stage microfluidic arrays have typically been able to handle only very dilute blood samples, at very low volumetric flow rates, with relatively poor cell separation performance.[24, 25] Devices employing magnetic separation would be prohibitively enormous and costly, as well as incapable of processing platelets.[26] Potential “low tech” user-powered methods (e.g. hand crank devices) are characteristically labor-intensive and subject to human error and/or mechanical failure.[27]

In this study, we present a proof-of-concept prototype of a new dual-stage, passive separation system for processing donated WB into components, which does not require costly or complex machinery, and can be used on-site by minimally-trained personnel immediately following blood donation. This approach consists of two passive blood-processing modules, neither of which requires electricity: (i) a blood-bag compression apparatus designed to maximize the speed and efficiency of passive RBC sedimentation for separating RBCs and platelet-rich plasma (PRP) from WB at normal gravity (i.e. 1×*g*), and (ii) a flow-through microfluidic concentrator for continuous high-throughput enrichment of platelets from the PRP fraction, to produce PC and purified plasma. The ability of this technology to produce high-quality blood components quickly without the use of a centrifuge is demonstrated, and its potential utility in resource-limited settings lacking a robust blood banking infrastructure is discussed.

## Materials and methods

### Fabrication of the passive WB separation system

The disposable kit of the passive WB separation system was constructed by sterilely connecting a 1 L transfer bag (T3107; Charter Medical, Winston-Salem, NC), used for RBC sedimentation, via a valve (D100-455980, Braun Medical, Bethlehem, PA) to various standard components (e.g. storage bags, tubing, RBC leukoreduction filter) taken from a commercially-available storage system (123–94; Haemonetics Corporation, Braintree, MA), and the microfluidic platelet concentrator (MPC) fabricated using soft lithography (see below).

The custom compression apparatus used to express separated PRP after sedimentation consisted of a machined aluminum ring (8” O.D., 6” I.D., 6061) enclosing two parallel compression plates and an air spring (1B5-500; Goodyear, Akron, OH) for pushing the top plate against the bottom plate, through the sedimentation bag placed in between. A pressure gauge (595–06; Ashcroft, Stratford, CT) was mounted on top of the ring to monitor the pressure in the air spring, and the whole assembly was placed on a circular stand (7” O.D., 5” I.D.) that allowed for adjusting the angle between the plane of the sedimentation bag / compression plates and the direction of gravity. The dimensions and positioning of each part of the custom expressor were chosen to ensure that the air spring would be in the quasi-constant range of its force/extension curve throughout the PRP expression process (i.e. from ~10 mm to ~5 mm). A tube connecting the sedimentation bag with the rest of the disposable kit components was positioned to align with a notch in the top plate of the expressor, to allow for expression of separated PRP from the uppermost point of the top internal face of the bag.

The MPC module used to separate PRP into PC and PPP is based on the principle of controlled incremental filtration (CIF), which has been described previously in detail.[28, 29] Here the design of a CIF-based microfluidic device was optimized to allow flow-through concentration of platelets (typically 1.5–4 μm in diameter), while limiting the minimal feature size to no smaller than ~20 μm in width/spacing. This minimal feature size design criterion ensures that channels can be fabricated that are ~140 μm in depth (via standard techniques), and therefore that meaningful volumetric flowrates may be achieved at a relatively low driving pressure (here, ~10 PSI). Each device was designed to syphon platelet poor plasma (PPP) from the flowing PRP suspension, until the platelet count of the progressively-concentrated PRP was increased by ~3×. Ten individual platelet concentration CIF devices were patterned into a parallelized array, with appropriate in-plane connections made to allow for output ports for PC and PPP effluent streams, and were designed to fit onto a 4” (10 cm) circular mold.

Chrome-on-glass photomasks (Photo Sciences, Inc., Torrance, CA) were generated from the CAD drawings of the MPC module and used to create corresponding relief structures in SU8 photoresist (Microchem, Westborough, MA), which had been spin-coated onto a 4” silicon wafer (University Wafer, South Boston, MA). Additional masters were created to serve as molds for a ‘top layer’ to the module, which consisted of wide feed channels to distribute the incoming PRP to the individual CIF devices in the two ‘device layers’ bonded beneath it. Elastomeric polydimethylsiloxane (PDMS; SylGard 184, Dow Corning Corp, Midland, MI) casts were cut and peeled from the SU8-on-silicon masters. Appropriate through-holes were made in each PDMS layer using biopsy punches (Acuderm, Fort Lauderdale, FL), and the four components of the module (top layer, two device layers, and a flat base) were plasma oxidized (Plasmalab 80 Plus, Oxford Instruments, Abingdon, United Kingdom) and sealed together.[30] Sealed MPC modules were wet with a 1% (w/v) mPEG (MW 5000, Laysan Bio Inc, Arab, AL) solution in deionized water, followed by incubation in a 1% human serum albumin solution in PBS. Appropriate tubing, which later would be welded to standard blood/platelet storage bags, was inserted using barbed connectors, heat-sealed on the opposing end, and the entire module was sterilized by exposure to high intensity UV light for 90 min (TC312E, Spectroline, Westbury, NY). Fabricated MPC modules were kept at 37°C for a minimum of 5 days and inspected for presence of bacteria to ensure sterility prior to use.

### Blood collection

Units of blood (n = 6) from healthy donors who met AABB/FDA eligibility criteria for volunteer blood donation were randomly selected from blood collected at the MD Anderson Cancer Center Blood Donor Center (Houston, Texas, USA), with written informed consent under an IRB approved protocol (PA15-0230). Each unit was collected as whole blood (~500 mL) into a standard blood collection bag containing 70 mL citrate phosphate dextrose (CPD) as anticoagulant (Fenwal, Lake Zurich, IL), and then transported to the University of Houston for processing within one hour of donation.

### Study design

A dual-arm split-unit study was conducted to compare the initial quality of packed RBCs, platelet concentrate, and plasma components produced with a new passive separation system (test arm) versus traditional centrifugation (control arm). Each of the donated WB units (mean volume with anticoagulant 562 ±20 mL, range: 535–585 mL) was split into two samples of 250 ±5 mL via sterile connections and fully processed within four hours of donation. Key biochemical and rheological parameters of the final products produced by each technique were then measured for comparison.

### Separation using conventional centrifugation protocol

The control arm 250mL WB sample was first allowed to cool to room temperature (~22°C), then separated into RBCs and PRP by so-called ‘soft spin’ centrifugation (1780×g for 310 sec; Allegra X-15R; Beckman Coulter Inc., Brea, CA). The supernatant PRP was expressed into an attached bag using a standard plasma expressor (Teruflex ACS-201; Terumo, Elkton, MD) and then ‘hard spun’ (4200×g for 350 sec) to sediment platelets. The supernatant plasma (~90 mL) was then expressed and the residual platelet concentrate (~30 mL) was allowed to sit for 1 hour prior to sampling, following standard U.S. blood banking procedures.[1, 7] AS-3 additive solution (55 mL; Haemonetics Corporation) from an attached RBC storage bag was added into the packed RBC sample bag via a sterile connection, mixed, and the mixture was then leukocyte reduced via a leukocyte reduction filter (High Efficiency Leukocyte Reduction Filter; Haemonetics Corporation), which also removes residual platelets, and collected in the pRBC storage bag. The individual pRBC, PC, and PPP bags were heat sealed (TCD B40; Genesis BPS, Ramsey, NJ) and disconnected from the blood collection set, before being fitted with spiked ports for syringe sampling in a sterile hood.

### Separation using the dual-stage, passive separation system

The test arm 250 mL WB sample was collected into a 1 L blood transfer bag and placed between the two plates of a custom-made compression apparatus, at a slightly inclined angle (~10°) to facilitate expression of PRP following sedimentation of WB at unit gravity. After 150 minutes of natural sedimentation, the supernatant PRP was expressed from the sedimentation bag and through an attached microfluidic platelet concentrator (MPC). PRP was separated into PC and platelet poor plasma (PPP) as it flowed through the MPC device at ~3.2 mL/min. Total separation time (sedimentation plus PRP processing) ranged from 172 to 195 minutes. Output streams from the MPC device were collected directly into the same type of storage bags used for centrifugation-produced PC and PPP components. Once PRP expression had completed, the sedimented RBCs were mixed with AS-3 solution and leukoreduced in the same manner as centrifugation-produced pRBC components. The entire separation process for a 250 mL WB half-unit was completed within ~3 hours.

### Measurements of blood component quality

A 16-parameter complete blood count with 3-part differential (including hematocrit, mean corpuscular volume, platelet count, mean platelet volume, and white blood cell count) was performed for all samples using an automated hematology analyzer (Medonic M16M/M20MGP Open Vial; Clinical Diagnostic Solutions, Inc., Plantation, FL). Platelet P-selectin expression, RBC PS exposure, and platelet PS exposure were measured via flow cytometry (FACS Aria II; BD Biosciences, Franklin Lakes, NJ) using commercially-available kits according to manufacturers’ instructions. Level of free hemoglobin (Hb) was measured for WB and pRBC samples spectrophotometrically, using modified Drabkin’s method (SpectraMax M5; Molecular Devices Inc., Sunnyvale, CA). Percent hemolysis was calculated as % hemolysis = [supernatant Hb] * [100—% hematocrit] / [total Hb]. RBC deformability was measured for WB and pRBC samples using an ektacytometer (LORRCA; Mechatronics, Zwagg, The Netherlands). Biochemical parameters (including pH, K^+^, Na^+^, blood urea nitrogen, creatinine, partial pressure of oxygen (PaO_2_), partial pressure of carbon dioxide (PaCO_2_), oxygen saturation, total carbon dioxide content, glucose, and lactate) were measured using a handheld blood analyzer with corresponding cartridge inserts (iSTAT; Abbott Laboratories, Abbott Park, IL). The level of ATP was measured using a bioluminescent somatic cell assay, and 2,3-DPG levels were measured a standard UV assay (Sigma-Aldrich, St. Louis, MO). Platelet aggregability was measured using two physiologically-relevant strong agonists (thrombin receptor agonist peptide, TRAP, and adenosine diphosphate, ADP), and a weak agonist (collagen) with an aggregometer (PAP-8E; Bio/Data Corp, Horsham, PA). Commercially-available enzyme-linked immunosorbent assays (ELISAs) were used to determine the levels of coagulation factors VIII and XI (Affinity Biologicals, Hamilton, Ontario, Canada) and the levels of soluble CD40-ligand (sCD40L; eBioscience, Vienna, Austria) and thromboxane B2 (TxB_2_; Cayman Chemicals, Ann Arbor, MI). Total free protein was measured using a Pierce^™^ 660 nm protein assay kit (Fisher Scientific, Pittsburgh, PA). Necessary absorbance measurements for each assay were made using a plate reader (SpectraMax M5; Molecular Devices, Sunnyvale, CA).

### Statistical analysis

Results are expressed as mean values ± standard deviation, or mean values ± standard deviation (minimum—maximum). Paired two-sided t-tests were performed to compare the data acquired on blood products manufactured from the passive WB separation system and from conventional centrifugation. Significance was defined as p < 0.05 for pRBC parameters and p < 0.01 for PC and plasma parameters, due to the inherent higher volatility of platelet and plasma factors between subjects.

## Results

### Design and operation of the passive WB separation system

**Fig 1:**
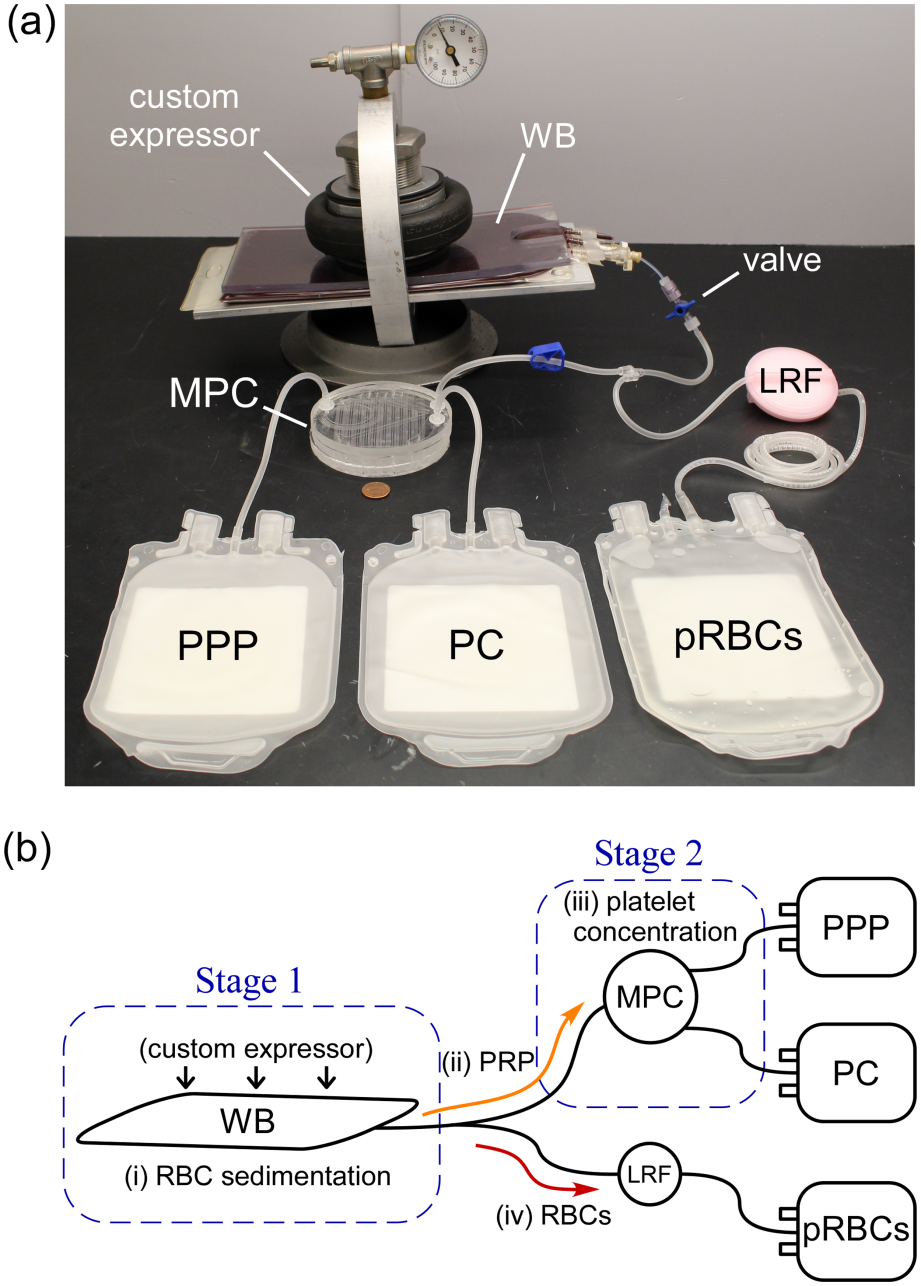
The passive, centrifugation-free system for separating WB into components. (**a**) Photograph of the WB separation system, including the custom compression/expressor apparatus and components of the disposable kit. A one cent U.S. coin is shown for size reference. (**b**) A schematic illustration of the system’s operation: (i) WB is separated into PRP and RBCs via natural sedimentation in a shallow blood bag held within a custom compression/expressor apparatus, (ii) PRP is expressed from the sedimentation bag, (iii) PRP passing through the microfluidic platelet concentrator (MPC) is separated into PC and PPP, and (iv) sedimented RBCs are mixed with additive solution and expressed from the sedimentation bag through a standard leukoreduction filter (LRF).

Fig 1 shows the passive WB separation system (Fig 1a) and illustrates its overall operation schematically (Fig 1b). The system has two primary modules, one is designed to separate WB into pRBCs and PRP via natural sedimentation of RBCs at normal gravity (Stage 1; Fig 1b), and the other is designed to separate PRP into PC and PPP using novel high-throughput microfluidic technology (Stage 2; Fig 1b).

**Fig 2:**
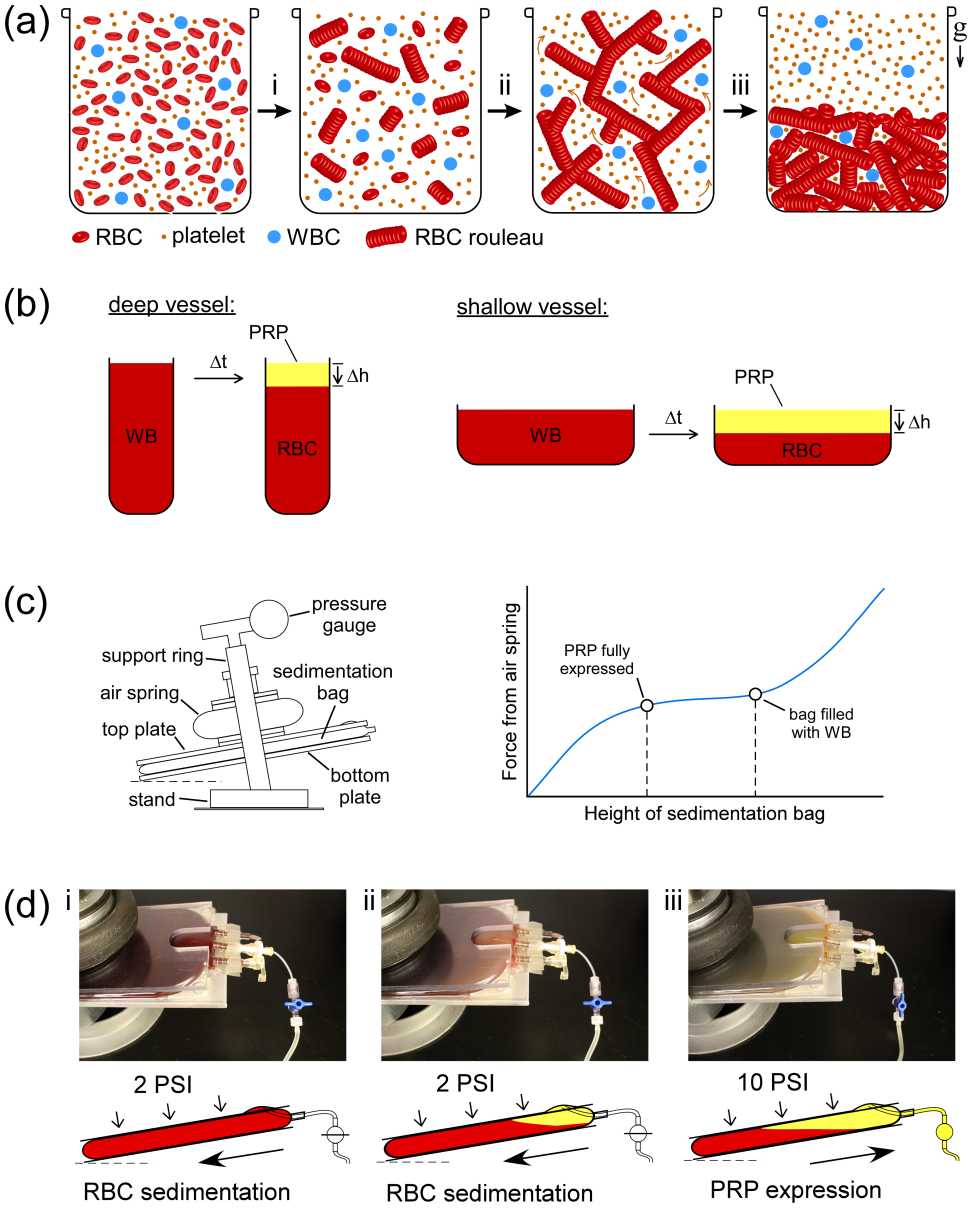
Separation of WB into RBCs and PRP via RBC sedimentation (Stage 1). (**a**) Schematic illustration of the process of natural RBC sedimentation at 1×g gravity: (i) RBCs form rouleaux that (ii) gradually assemble into a large network, which then (iii) collapses as PRP ‘channels’ upward through the network of RBC aggregates falling towards to the bottom of the vessel. (**b**) For a given erythrocyte sedimentation rate (ESR = Δh/Δt), a WB sample in a shallow vessel achieves a greater separation of PRP and RBCs, in a given amount of time. (**c**) The custom compression/expressor apparatus, utilizing the constant force regime of an air spring to provide a consistent flow rate through the platelet concentration module. (**d**) Separation of RBCs from PRP via passive sedimentation over time: i) 250 mL of freshly-drawn WB in a 1 L sedimentation bag, placed on a slightly (10°) angled base plate and gently compressed by an air spring (~2 PSI); ii) within ~60 min the ‘shallow pool’ of WB within the sedimentation bag has largely separated into pRBCs and PRP; iii) after 150 min, the pressure in the bag is increased to ~10 PSI, and the exit valve is opened, expressing the PRP layer through the Stage 2 microfluidic platelet concentrator (MPC) downstream (see Fig 3).

Fig 2 illustrates the Stage 1 separation of WB into RBCs and PRP. Before beginning to sediment at unit gravity (1×g), RBCs undergo a waiting period during which they weakly aggregate to form a large-scale network. This aggregation proceeds first by so-called face-to-face ‘rouleau’ formation, and then via formation of much larger multi-rouleaux face-to-side conglomerates (note that platelets are largely excluded from these structures) (Fig 2a). Once RBC aggregation propagates, plasma begins to flow through the porous RBC network from bottom to top, carrying with it the majority of platelets (Fig 2a). This ‘channeling’ of PRP up through the RBC network causes the RBC aggregates to collapse into a packed layer (Fig 2a).[31, 32] In both deep and shallow vessels, this process of RBC sedimentation proceeds at approximately the same rate (the so-called erythrocyte sedimentation rate, ESR), for a given blood sample, which is normally ≤ 20 mm/hr for men and ≤ 30 mm/hr for women.[33] That is, once formed, the interface between the PRP layer and RBC layer of a sedimenting WB sample will drop a similar height (Δh) in a given amount of time (Δt), even in different aspect ratio vessels (Fig 2b). Therefore, the *volume* of PRP that is yielded by RBC sedimentation over a period of time in a shallow vessel is greater than for the same volume of WB contained in a deeper vessel (Fig 2b). We utilized this natural phenomenon to develop an electricity-free approach for separating a large volume of WB into RBCs and PRP at 1×g, without the need for centrifugation, by essentially spreading WB into a wide, but shallow (8–10 mm) layer within an oversized (sedimentation) bag, compressed by a specially designed expressor.

The Stage 1 module of our separation system consists of a simple custom-made apparatus (Fig 2c) that is designed in such a way as to provide a quasi-constant amount of force on the sedimentation bag of the passive WB separation kit throughout the PRP expression process (Fig 2c). When the sedimentation bag is placed between the compression plates after being filled with the desired amount of freshly donated WB, the air spring is initially inflated to 8 PSI (55.16 kPa) to push against the top plate of the apparatus, creating enough pressure within the bag (~2 PSI, or 13.79 kPa) to provide it with a stable structural form (Fig 2d). In a sedimentation bag that is sufficiently large to keep the ‘height’ of the WB sample at 8–10 mm high, full sedimentation of the RBCs occurs within ~1–2 hrs, depending on the characteristics of the donated WB, however 2.5 hrs was chosen as a standard sedimentation timeframe in this study as a factor of safety (i.e. to still function effectively, even for donors with exceedingly slow-sedimenting RBCs).

During sedimentation, the entire Stage 1 apparatus was tilted on its base slightly (~10°, Fig 2c) to ensure the PRP supernatant of the biphasic WB solution could be fully collected following sedimentation. After 150 minutes of passive RBC sedimentation, the pressure in the air-spring is increased to 40 PSI (275.79 kPa) to pressurize the sedimentation bag to ~10 PSI (68.95 kPa), and the valve on the tubing connecting the sedimentation bag with the rest of the disposable kit is opened to initiate expression of the PRP layer that has formed in the top of the bag (Fig 2d). (Note that the air spring can be easily inflated to the range of operating pressures used in our application with a standard, hand-powered bicycle pump.) Once the packed RBC layer begins entering the microfluidic module, the flow rate slows ‘automatically’ due to the dramatically higher viscosity, and PRP expression is manually stopped.

**Fig 3:**
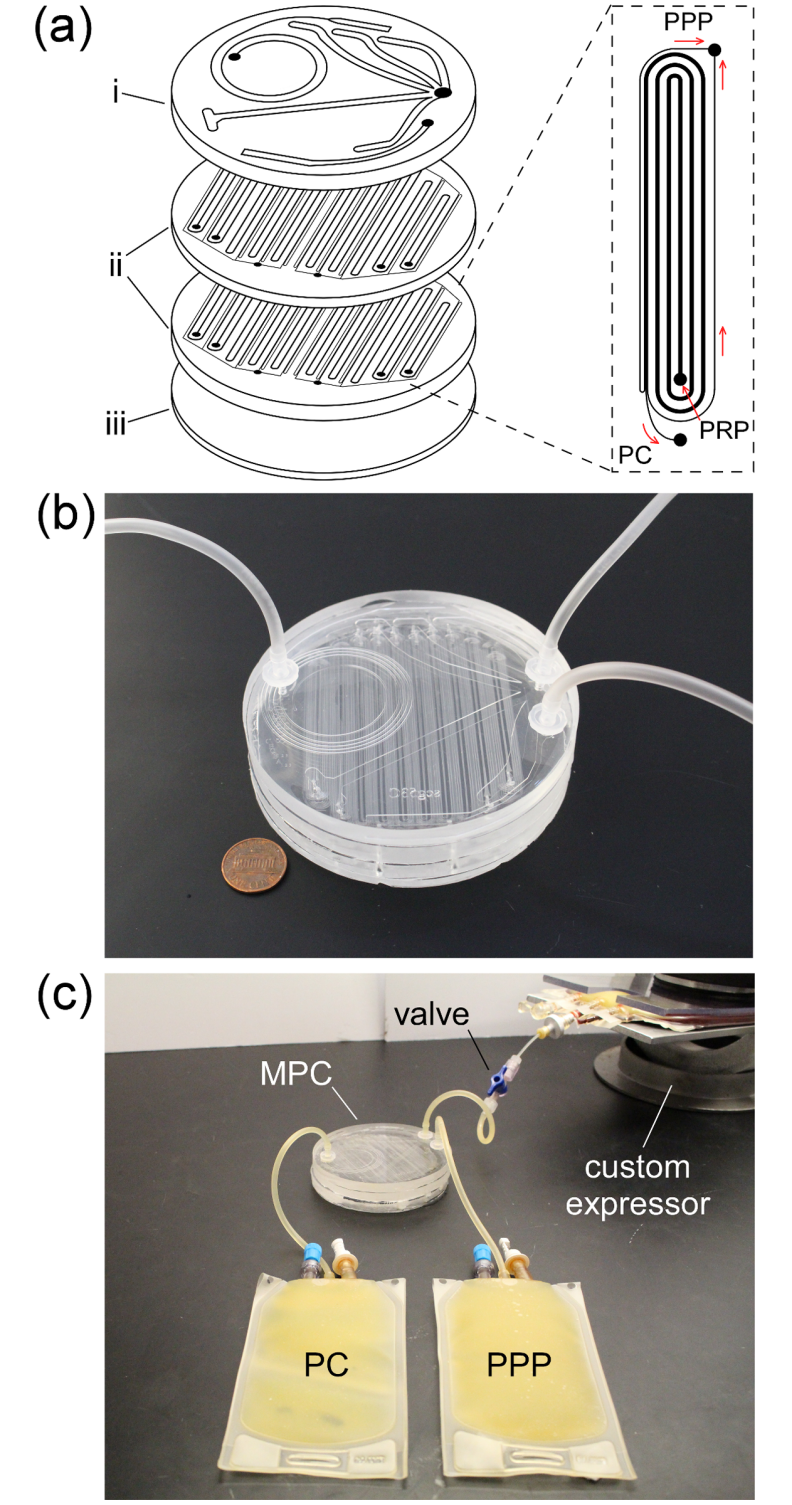
The microfluidic platelet concentrator (MPC) of passive WB separation system (Stage 2). (**a**) Components of the MPC: (i) top layer, containing five inlet channel branches for distributing PRP throughout the device, and two outlet channels for collecting the streams of separated PC and PPP; (ii) two device layers each consisting of ten individual CIF microfluidic devices in parallel, which perform the separation; (iii) a flat bottom layer for sealing the device. Inset shows a single CIF microdevice schematically; arrows indicate the direction of flow. (**b**) Photograph of an assembled MPC device. A one cent U.S. coin is shown for size reference. (**c**) Photograph of PRP being expressed from the sedimentation bag, through the MPC module, separated into PC and PPP.

The Stage 2 module of the passive WB separation system consists of the microfluidic platelet concentrator (MPC, Fig 3) connected to the compressed sedimentation bag via tubing that is valved off during sedimentation (Figs 1a and 2d). The relatively modest pressure within the sedimentation bag during expression drives the flow of PRP (at ~3.2 mL/min) through the MPC module, which continuously separates the PRP into platelet concentrate (PC) and plasma (PPP) (Fig 3). The design of the MPC module is based on recently-developed ‘controlled incremental filtration’ (CIF) technology, which enables high-precision separation of particles by size at high volumetric throughputs in specially-designed microfluidic channels with reasonably large minimal feature sizes.[28, 29] Each MPC module consists of several CIF-based microfluidic devices connected in parallel and fabricated using standard soft lithography techniques (Fig 3a and 3b). Separated PC and PPP outflow from the MPC module directly into their respective storage bags (Fig 3c).

Finally, after RBC sedimentation has completed and the separated PRP has been fully expressed from the compressed sedimentation bag through the MPC module, generating PC and PPP, the sedimented RBCs are mixed with AS-3 storage solution and leukoreduced (Fig 1b).

### Comparison of the passive WB separation system with standard centrifugation protocol

**Fig 4:**
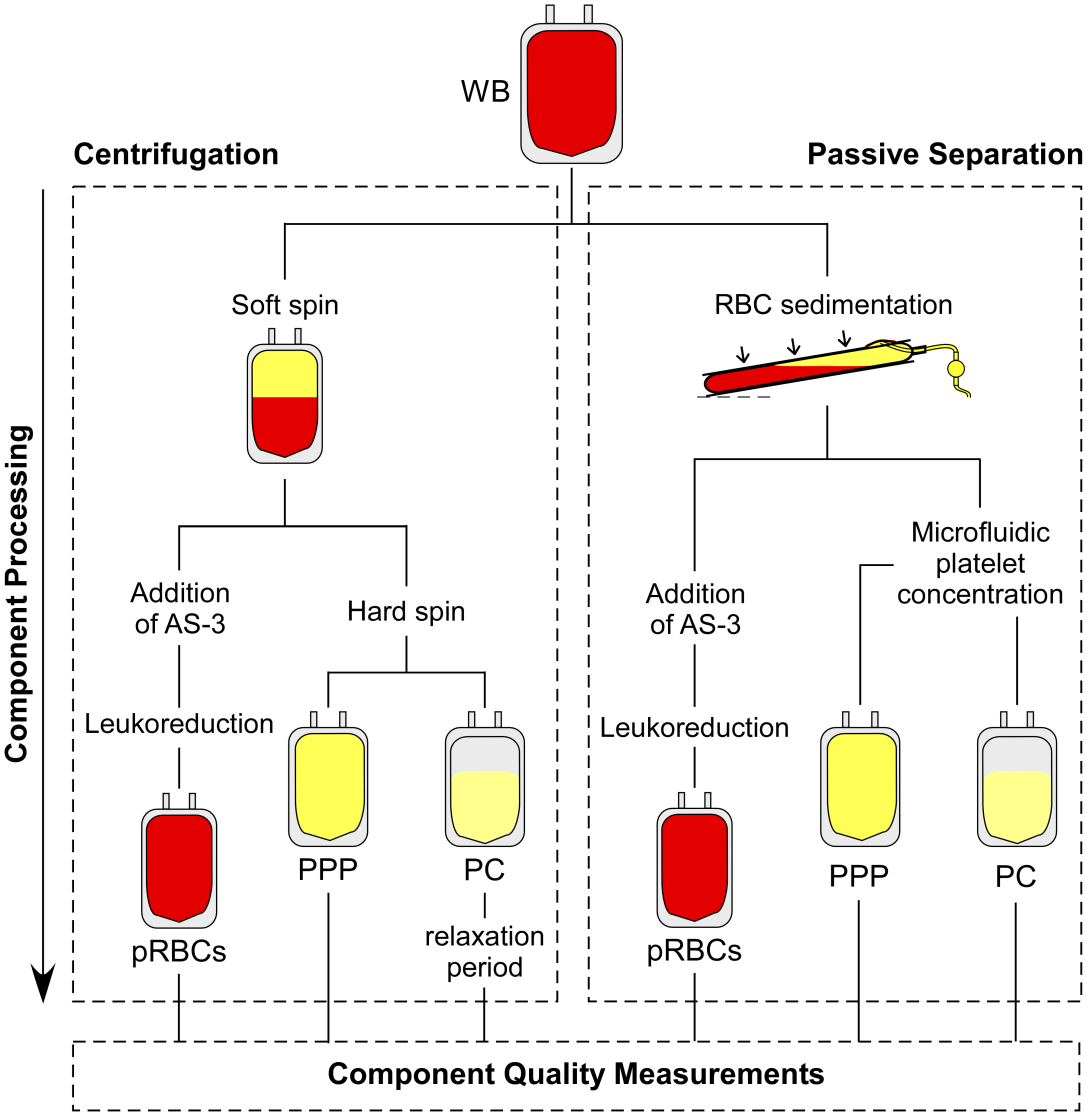
Schematic illustration of the design of the dual arm, split unit study comparing the quality of blood components produced using the new passive separation system versus conventional centrifugation.

We have conducted a dual-arm split-unit study to compare the initial quality of units of packed RBCs (pRBCs), platelet concentrate (PC), and platelet poor plasma (PPP) produced with the new passive WB separation system (test arm) versus those manufactured via traditional centrifugation (control arm) (Fig 4). Each unit of freshly donated WB was split into two equal 250 mL “half-units” via sterile connections, and processed within four hours of donation. The half-unit in the control arm was processed into components via the so-called ‘PRP method’ commonly used in the U.S.,[1] and the half-unit in the test arm was processed using our new dual-stage, passive WB system (Fig 1). We measured a broad panel of hematological parameters for RBCs, platelets, and plasma (Table 1) immediately before and after separating the donated WB into components, for both separation methods. The 2,3-diphosphoglycerate (2,3-DPG) for the passively-separated RBCs (15.79 ± 1.24 μmol/g of Hb) was slightly, but statistically significantly (p = 0.03), higher than that for the centrifuged RBCs (15.27 ± 1.17 μmol/g of Hb). All other biochemical and rheological parameters for the pRBC products (HCT, hemolysis, PS exposure, ATP, lactate, glucose, sodium ion, pH, elongation index at 3 and 30 Pa measured using ektacytometry) did not show significant (p < 0.05) differences between the two separation methods (Table 1). The higher amount of pRBC unit volume (and lower HCT) observed for the passive separation method indicates that red cell packing density is lower when unit gravity is used to sediment RBCs, rather than high-speed centrifugation, as expected.

**Table 1:** 

	Whole blood	Centrifugation	Passive separation
**RBCs / pRBC unit**			
Volume [mL]	561.7 ± 19.9	178.0 ± 14.2*	198.9 ± 25.4*
WBC [10^3^/μL]	4.69 ± 1.00	0 ± 0^#^	0 ± 0^#^
Platelet Count [10^3^/μL]	160 ± 29	0 ± 0^#^	0 ± 0^#^
Hematocrit [%]	33.8 ± 3.4	43.7 ± 3.5	40.2 ± 3.2
Hemolysis [%]	0.21 ± 0.11	0.09 ± 0.07	0.09 ± 0.04
Hemoglobin [g/dL]	11.5 ± 1.0	13.9 ± 1.2	12.8 ± 0.6
PS Exposure [%]	0.24 ± 0.07	0.11 ± 0.05	0.12 ± 0.05
ATP [μmol/g of Hb]	4.37 ± 0.19	4.29 ± 0.19	4.40 ± 0.18
2,3-DPG [μmol/g of Hb]	16.01 ± 1.18	15.27 ± 1.17*	15.79 ± 1.24*
Lactate [mmol/L]	2.63 ± 0.29	1.91 ± 0.24	1.91 ± 0.31
Glucose [mg/dL]	364.0 ± 13.3	523.7 ± 19.3	514.8 ± 24.3
Sodium [mmol/L]	143.8 ± 1.6	134.3 ± 1.2	135.0 ± 1.1
pH	7.020 ± 0.051	6.808 ± 0.063	6.826 ± 0.057
Elongation Index [3 Pa]	0.384 ± 0.017	0.387 ± 0.006	0.391 ± 0.015
Elongation Index [30 Pa]	0.609 ± 0.008	0.612 ± 0.005	0.610 ± 0.006
**Platelets / PC unit**			
Volume [mL]	-	30.1 ± 6.1	29.5 ± 6.8
WBC [10^3^/μL]	-	4.78 ± 1.82*	0.35 ± 0.38*
Platelet Count [10^3^/μL]	-	1151 ± 383	873 ± 146
Platelet Recovery (%)	-	96.20 ± 2.73*	87.48 ± 4.31*
P-Selectin (%)	2.65 ± 1.38	12.23 ± 3.61*	5.22 ± 2.14*
PS Exposure (%)	1.28 ± 0.60	3.30 ± 1.94	1.35 ± 0.72
pH	-	7.313 ± 0.093	7.382 ± 0.060
Hemoglobin [g/dL]	-	0.1 ± 0.1	0.1 ± 0.1
TRAP^†^ (AUC)	-	17618 ± 615	18711 ± 1248
ADP^†^ (AUC)	-	16477 ± 817	16853 ± 904
Collagen^†^ (AUC)	-	68291 ± 3206	72083 ± 3577
Plasma sCD40L [ng/mL]	-	1.761 ± 0.306*	1.567 ± 0.284*
Plasma TxB_2_ [ng/mL]	-	0.473 ± 0.123*	0.416 ± 0.105*
**Plasma / PPP unit**			
Volume [mL]	-	85.0 ± 9.5	69.8 ± 18.6
WBC [10^3^/μL]	-	0.01 ± 0.01	0.01 ± 0.01
Platelet Count [10^3^/μL]	-	12 ± 11*	52 ± 21*
Hemoglobin [g/dL]	-	0 ± 0^#^	0 ± 0^#^
Total Protein^†^ [mg/mL]	-	44.94 ± 11.38	51.76 ± 11.78
Factor VIII [IU/mL]	-	1.357 ± 0.427	1.274 ± 0.405
Factor XI [IU/mL]	-	0.874 ± 0.229	0.874 ± 0.149

Key RBC, platelet and plasma parameters for donated WB, and blood components (pRBC, PC and PPP units) produced via conventional centrifugation or using the passive separation system. Values are mean ± standard deviation (n = 6; † indicates n = 5 due to data acquisition failure);

^#^ indicates a measurement below limit of detection for our hematology analyzer;

* indicates statistical significance (p < 0.05 for RBCs / pRBC units, p < 0.01 for platelets / PC units and plasma / PPP units; two-tailed paired t-test).

Several key parameters for the platelet and plasma units (Table 1) showed statistically significant differences at a higher significance threshold (p < 0.01). The MPC module of the passive WB separation system retained 87.48 ± 4.31% of platelets from the PRP when producing a PC unit, while centrifugation was able to recover 96.20 ± 2.73% of platelets (p = 0.002). P-selectin, a surface marker for platelet activation, was expressed in 5.22 ± 2.14% of platelets within PC units produced using the passive WB separation system, while units prepared using the standard centrifugation-based method had 12.23 ± 3.61% P-selectin expression (p = 0.005). In addition, the concentration of residual platelets in passive system-derived plasma was significantly higher, and the concentration of WBCs in passive system-derived PC was significantly lower, than in their centrifugation-derived counterparts.

**Fig 5:**
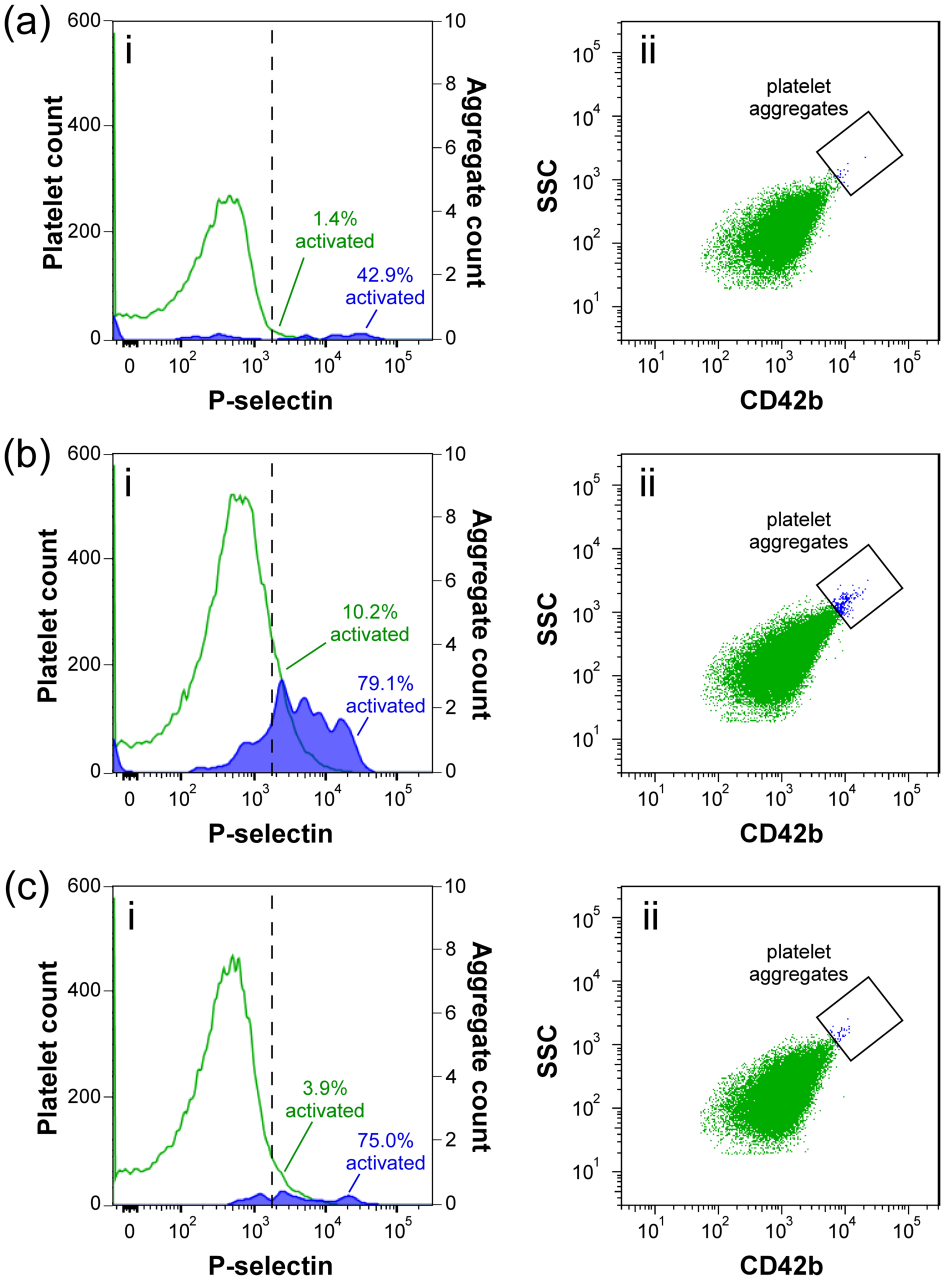
Activation of platelets due to processing of a typical WB unit into components using the two separation methods. Each panel shows i) the level of platelet activation (primary y-axis: all platelets, green; secondary y-axis: platelet aggregates, blue), and ii) the associated formation of platelet aggregates, for (**a**) the WB unit prior to processing, and the two PC units produced (**b**) via conventional centrifugation, and (**c**) using the passive separation system. Platelets were labelled with CD42b-PerCP, and activated platelets were identified using CD62p (P-selectin)-FITC. The threshold of positive P-selectin expression was defined using an isotype control (IgG-FITC). A manual gate was applied to SSC vs. CD42b scatter plots to demarcate the presence of any (typically highly activated) platelet aggregates, which would be observed in the form of a characteristic ‘comet tail’ near the upper-right edge of a platelet distribution.

Fig 5 shows the impact of the separation method on platelet quality for a typical processed blood unit. The PC unit that was produced using centrifugation (Fig 5b) was significantly more activated and contained more platelet aggregates than the unit produced using the passive separation system (Fig 5c). This higher level of platelet activation following centrifugation was also reflected in the significantly higher levels of undesirable pro-inflammatory mediators released by platelets into the plasma during processing—sCD40L (p = 0.002) and TxB_2_ (p = 0.003)–that we measured in the PC units produced by centrifugation (Table 1).

The ability of platelets to aggregate is a valuable measurement in assessing platelet function, therefore we measured platelet aggregability with physiologically-relevant strong (TRAP, ADP) and weak (collagen) agonists (Table 1). For all three agonists, in every paired sample set studied, the platelet aggregability in the PC unit from the passive separation system was superior to its centrifugation counterpart. Likewise, every measurement of pH and PS exposure made for PC units produced by passive separation compared favorably to those measured in the paired PC units produced by centrifugation. Larger sample sizes would be required, however, to make a more definitive assessment of whether these observed trends in aggregability, pH, and PS exposure will prove to be statistically significant.

## Discussion

The newly-developed approach to blood processing described in this report challenges the current paradigm that centrifugation of WB is the only efficient means to fully separate donor blood into the three classical transfusion products (pRBCs, PC, and PPP). This novel system has several demonstrated (and innate) advantages over conventional centrifugation.

### Logistical advantages

The Stage 1 sedimentation step of the portable WB separation system typically takes 90–150 minutes, depending on the donor, while the prototype Stage 2 MPC can separate 100mL of PRP in ~30 minutes (at ~10 PSI driving pressure), thus, the separation process for a full (~500mL) unit of WB could be completed within 2.5–3.5 hours using a similar approach. This time requirement will be reduced with further system improvements, however it is important to note that with current practices it is often hours before donated WB units reach a blood processing center to undergo centrifugation.[8, 34] Blood processing using our technology can instead commence immediately after blood donation, and therefore may well complete in the time it would take to simply transport collected WB units to a centralized blood processing facility, even with no further improvements to the prototype system. In contrast to centrifugation, all collected WB units could also be processed simultaneously (e.g. a typical centrifuge can process only 4 to 6 units at a time, and therefore will be continuously backlogged during times of higher demand), and will be subjected to less opportunity for human error (which may increase during the night shift processing that many blood banks must employ to handle WB donations collected during the day).[6]

The fact that separation using our new system can be performed with nothing more than an inexpensive compression apparatus (total cost of materials and custom machining, to fit compression plates and air spring securely within the support ring: < 200 USD), a 4” (~10 cm) plastic module (which can be injection molded or hot embossed using existing manufacturing methods), and standard off-the-shelf blood collection/storage bags, implies that this system could be an attractive solution for use in a variety of resource-limited, austere environments that lack the expensive (and largely immobile) equipment needed in conventional blood processing. The entire system can be contained in ~ 2 sqft (0.19 m^2^) of bench space, and, as the air spring pressures required are 40PSI (275.79 kPa) or lower (to produce ~10 PSI within the sedimentation blood bag), it can be driven using a simple hand- or bicycle-pump (i.e. electricity is not necessary). In addition, even the inexpensive compression/expressor apparatus could be eliminated, and simple hydrostatic pressure used to drive flow of the separated PRP through the MPC module, if the processing of donated blood were to be performed under less stringent ‘overnight hold’-style conditions.[6]

The design of the MPC module would also readily allow for the ‘washing’ of platelets into additive solution prior to storage (which would be beneficial particularly for cold-stored PC),[35, 36] and/or the removal of waste-product-rich media from the PC unit post-storage and prior to transfusion.[37, 38] Further, its physical format and microchannel-based architecture could enable a straightforward (UV-based) pathogen inactivation strategy to be employed simultaneously as platelets were being concentrated by the device.

The low cost and portability of this new approach could help developing nations realize a robust blood banking infrastructure—once the progression from direct WB transfusion to more efficient component therapy has been initiated, as well as improve existing capacity for industrialized societies to maintain blood component supplies in remote/rural healthcare facilities, at times of man-made or natural disasters, and across the fragmented battlefields of modern armed conflict in remote areas of the world.

### Component quality advantages

High-speed centrifugation of WB invariably imposes significant forces onto RBCs, resulting in minor hemolysis, but potentially more extensive sub-hemolytic damage.[18, 39–41] During the subsequent storage period, any injurious effects from the initial mechanical stress on the RBCs cannot be recovered by adaptive cellular repair, since they lack a cell nucleus and most organelles.[42] Thus, cellular damage progressively accumulates into the so-called red blood cell ‘storage lesion,’ a combination of morphological and biochemical deterioration during storage.[43–45] Recently, it has been shown that the release of iron from RBCs—invariably damaged by standard processing and storage—may cause inflammation and increased mortality in transfusion recipients.[17, 46] Given the higher level of 2,3-DPG (a key factor for maintaining the oxygen delivery function of transfused blood) observed in RBC units produced with the passive system, it will be of great interest in future studies to test these units through their entire storage duration, and observe if the gentle sedimentation approach to initial processing may serve to lessen the onset and/or severity of the RBC storage lesion.

High-speed centrifugation is more clearly harmful to platelets, as they are inherently activated by shear stress and compaction/pelleting.[47, 48] The two stages of conventional WB processing (either soft-spin/hard-spin as in the ‘PRP method,’ or vice versa in the ‘buffy coat method’) are well known to trigger platelet activation (as assessed, for example, by transfer of P-selectin or phosphatidylserine to their outer surface), which by definition quickly compromises their structural integrity—as well as their efficacy following future transfusion.[49–52] Skripchenko et al. showed that P-selectin expression increases in some cases by ~40% after centrifugation of whole blood,[53] although in this study we were able to reduce the level of P-selectin expression from centrifugation to a more modest ~10% increase (i.e. from 2.7% to 12.2%, Table 1), by careful selection of our centrifugation protocol (see Materials and methods). Nevertheless, this represented a 4-fold larger increase as compared to platelets concentrated by our centrifuge-free system, only ~2.5% of which were activated above baseline levels (i.e. 5.22% P-selectin expression for PC units vs. 2.65% for WB).

Platelets activated *ex vivo* during WB processing have been shown to have shortened survival times post transfusion,[54] and minimal clinical value due to their severely diminished ability to aggregate.[55] Activated platelets, as part of their natural hemostatic function, will also release pro-activation molecules to recruit further participants into the clotting cascade.[56, 57] Thus, since the passive platelet concentrator used for this study resulted in a significantly lower increase in P-selectin expression and diminished release of inflammatory molecules sCD40L and TxB_2_ during initial processing (Table 1), it will also be of particular interest to monitor and compare the rate of increase in platelet activation during a 5–7 day storage duration of these PC units in future studies, as compared to conventional platelet units.

Full long-term storage studies of RBC, PC, and plasma units are currently being planned, and should help determine if the reduction in damage to RBCs and platelets produced by the new system during component processing will in fact become more amplified over time.

### Other performance comparisons

Our high-throughput microfluidic platelet concentrator retained 87.48 ±4.31% of platelets from the original PRP in the PC output stream of the module (Table 1; range: 79.40%–90.92%). Most of the platelets lost in this stage were the smallest fragments, as the MPV of the residual platelets in the plasma stream were consistently < 6fL (compared to 7.7–11.1 fL for the PC units). It is likely that many these fragments will not remain in the circulation for a significant amount of time following transfusion, and are therefore likely of limited long-term therapeutic value. Conventional centrifugation is nevertheless superior if the primary metric is simply bulk platelet recovery (96.20% ±2.73%; range: 92.44%–99.09%). However, the pelleting of platelets at high g-force, to maximize recovery, has the consequence of producing platelet aggregates in the PC unit (Fig 5). The absence of such aggregates from PC produced via the passive system implies that the sacrifice of platelet recovery may ultimately be a welcome tradeoff in order to achieve an overall higher quality product to be transfused into patients.

We also observed lower residual Hb content, as well as far fewer WBCs, in the PC units from the passive system (Table 1). While the traditional centrifugation approach is superior in terms of packing RBCs more tightly, the nature of the passive sedimentation approach (i.e. rouleaux formation; Fig 2) appears more effective in reducing WBCs in the PRP layer, likely due to WBCs being caught by the collapsing network of RBC aggregates during 1×*g* sedimentation. A much lower WBC count in PC units from the passive system (0.35 ± 0.38 K/μL) versus centrifugation (4.78 ± 1.82 K/μL), while not nearly as effective as complete leukoreduction (LR), could potentially imply a lower risk of developing refractoriness to PC transfusion as well as a number of other complications linked to the presence of WBCs in blood products.[58] Particularly in cases in which proper LR filtration of PC units is not feasible due to added cost, the significantly lower WBC count in PC units generated by passive separation may help mitigate some negative effects on both the platelets (during storage) and patients (following transfusion), which may be worthy of further study.

## Conclusions

This study demonstrated that high-speed centrifugation is not necessary to separate WB into high quality components nor is electricity. A half-scale prototype of an inexpensive, portable, and passive sedimentation / microfluidic system is able to viably compete with (and in some respects outcompete) the far costlier and much more laborious conventional methodology, in terms of both overall donation-to-storage time and quality of components. Once validated for processing full-scale blood units, this new technology could have a significant, positive impact on several stages of current blood donation and transfusion processes. Benefits include the potential for: (i) increasing access to more donors, thus augmenting the blood component supply, (ii) lowering operating expenses for blood collection centers and by extension reducing health care costs, and (iii) providing a higher quality product for patients, thereby reducing complications and risks associated with the transfusion of blood.

## Supporting information

S1 Table The minimal data set as a Microsoft Excel file. (XLSX)
